# Failed Attempts to Improve the Reliability of the Alcohol Visual Probe Task Following Empirical Recommendations

**DOI:** 10.1037/adb0000414

**Published:** 2018-11-26

**Authors:** Andrew Jones, Paul Christiansen, Matt Field

**Affiliations:** 1 Department of Psychological Sciences, University of Liverpool, and UK Centre for Tobacco and Alcohol Studies, Liverpool, United Kingdom

**Keywords:** alcohol, attentional bias, internal consistency, reliability, visual probe task

## Abstract

The visual probe task (VPT) is a computerized task used to measure attentional bias to substance-related stimuli. Little research has examined the psychometric properties of the VPT, despite concerns it demonstrates poor test–retest reliability and internal consistency. These issues can reduce confidence in inferences based on VPT performance. As such, we attempted to identify parameters under which the reliability of the alcohol VPT might be improved by applying recent empirical recommendations for outlier handling, bias calculation, and task design from the anxiety literature. We reanalyzed data from 3 previously published studies in our laboratory and 2 newly collected data sets. We compared tasks which presented images on the left/right of the screen to above/below, whether participants responded to the location or content of the probe, and whether general alcohol-related images or images personalized to the individual were used. In each VPT we also applied a priori outlier removal (2 and 3 standard deviations and median absolute difference) and data-driven outlier removal (winsorizing), in addition to calculating trial-level bias scores. Across all studies and tasks internal consistency and test–retest reliability of attentional bias measures were inadequate. There was no consistent improvement in internal consistency or test–retest reliability as a function of outlier removal methods. We were unable to demonstrate adequate reliability of the alcohol VPT, which further supports observations that these tasks may not yield reliable measures. Future research should focus on improving the reliability of these tasks or abandoning them in favor of more reliable alternatives.

Several theoretical models of addiction suggest that individuals who drink alcohol demonstrate preferential attention to alcohol-related cues in their environment, at the expense of competing cues (Franken, 2003; Robinson & Berridge, 2001). This preferential attention is often referred to as an “attentional bias.” Meta-analyses have demonstrated a small but robust link between attentional bias and craving (Field, Munafò, & Franken, 2009), and experimental manipulations of attentional bias have directly influenced alcohol consumption/relapse (Field & Eastwood, 2005; Schoenmakers et al., 2010) and craving (Luehring-Jones, Louis, Dennis-Tiwary, & Erblich, 2017) suggesting a possible causal relationship. However, more recently the clinical relevance of attentional bias has been challenged, with suggestions that weak findings are often overinterpreted and “null” findings ignored (Christiansen, Schoenmakers, & Field, 2015). Despite concerns, researchers continue to devote considerable effort to elucidating the exact role of attentional bias in addiction (and related behaviors such as obesity; Werthmann, Jansen, & Roefs, 2015).

One of the most popular tools used to measure attentional bias is the visual probe task (VPT, also known as the dot-probe task), first developed by MacLeod and colleagues (MacLeod, Mathews, & Tata, 1986). This task presents a pair of images: one alcohol-related and one control image (often a neutral or soft-drink image matched for composition and complexity). These images typically appear on the left- and right-hand side of the computer screen. Following a defined period, usually between 200–2,000 ms, these images disappear and a target probe appears in the spatial location previously occupied by one of these images. Participants have to make a response to the location or content of the probe as quickly as possible. If participants are faster to respond to the probes occurring in the space previously occupied by alcohol-related cues compared with a control cues, this is inferred as an attentional bias toward alcohol.

Despite widespread use and acceptance,^1^ there is much debate with regard to the reliability of the VPT for substances of abuse. Ataya et al. (2012) examined the internal reliability of several VPTs for alcohol and tobacco conducted in their laboratory and concluded internal consistency was poor (α = .00 to .50; mean .18). This supports more recent claims that the internal consistency of measures of cognitive biases are suboptimal and underreported (Parsons, 2018a). In response, we (Field & Christiansen, 2012) argued that the poor reliability may be due to specific features of the VPT, one of which was type of stimuli used in the task. Most studies provide a broad category of alcohol-related cues, however, these images may not represent the typical drinking habits of participants. For example, participants may identify as beer drinkers only, however during a VPT task they would be presented with stimuli depicting a broad range of alcoholic beverages (beer, wine, cider, spirits, etc.). To examine this, we (Christiansen, Mansfield, Duckworth, Field, & Jones, 2015) tailored a VPT to present only pictures that depicted the participants’ preferred drink category (e.g., beer-related cues) and demonstrated improved attentional bias compared with a more general category (α = .73 compared with α = .19). We also demonstrated that directly measuring attentional biases using eye-tracking technology increased internal consistency further for personalized images (α = .73), but also general images (α = .51).

As well as internal reliability, test–retest reliability (the consistency of a measure over time) is necessary for valid inferences from psychological tasks. This may be particularly important when attentional bias is measured repeatedly within individuals: for example, in the case of assessing changes in attentional bias that should arise after attentional bias modification interventions. Emery and Simons (2015) demonstrated that the VPT had poor split-half (*r* = −.19) and test–retest reliability (*r* = .13). Similarly in cocaine-using adults (Marks, Pike, Stoops, & Rush, 2014), test–retest reliability is low for reaction time (RT)-based measures (*r* = .24), but improved if examining eye movements (*r* = .51). Poor internal consistency and reliability threaten the validity of inferences that can be made using the VPT (Rodebaugh et al., 2016), and a failure to consider reliability might contribute to poor estimations of effect size and challenges to reproducibility (Parsons, 2018b; Zimmerman & Zumbo, 2015). Therefore, continued efforts need to be made to improve the psychometric properties of these tasks.

A recent paper attempted to provide empirical recommendations to improve the reliability of the VPT for anxiety-related images. Price and colleagues (2015) suggest that poor reliability of VPT may be due (in part) to how outlying RTs are handled when preparing the data for analyses. Typical procedures involve decisions based on cutoffs based on a valid response window for the population (e.g., RTs faster than 200 ms represent premature responding and slower than 2,000 ms suggest distraction), followed by removal of RTs which fall outside the distribution of the individual’s mean (e.g., 2 or 3 *SD*s). Research suggests that despite these techniques being the most popular method of removing outliers, they do not perform well under certain conditions (Leys, Ley, Klein, Bernard, & Licata, 2013) and there is little consensus across studies (cf., differing procedures are reported in each of these studies using alcohol VPT; Field & Powell, 2007; Miller & Fillmore, 2010; Townshend & Duka, 2001). Price et al. (2015) compared the reliability of bias scores following these outlier removal techniques with data-driven outlier removal in which outliers which fall outside of the observed distribution were rescaled (winsorized; Erceg-Hurn & Mirosevich, 2008). This procedure reduces the impact of outliers but also maintains all data points, increasing power. A further difference was how images were presented, in that standard alcohol VPT images are often presented on the left and right side of the screen, whereas Price et al. (2015) presented them at the top and bottom of the screen. They examined the effect procedural variables (probe location) may have on RT variance by examining the reliability on trials in which the probe only occurred in one position separately (e.g., bottom). Finally, they examined the reliability of bias scores averaged over tasks (given approximately 2 weeks apart), as an increased number of measures should increase reliability. To summarize, they found that test–retest reliability was greatest when (a) bias scores were calculated for probes that occurred behind the bottom image only, (b) winsorized outlier removal was used rather than arbitrary a priori cutoffs, and (c) data from repeated VPT were used, rather than a single task. The main focus of Price et al.’s (2015) investigation was the stability (test–retest) and internal consistency of attentional bias, unfortunately they did not consider internal consistency within the task(s) by examining bias scores on a picture-pair basis.

A second limitation of current data analytic techniques is the underlying assumption that attentional bias is a stable construct. This assumption is problematic because attentional bias may fluctuate within individuals during the course of the task (Zvielli, Amir, Goldstein, & Bernstein, 2016; Zvielli, Bernstein, & Koster, 2015, Iacoviello et al., 2014). For example, in deprived smokers attentional biases were evident only in phasic bursts within the VPT but were not evident when using the traditional overall (“global”) bias score. As such, we also calculated estimates of trial-level bias scores (TL-BS), based on recommendations by Zvielli et al., 2015).

Therefore, the aim of the current article was to apply the empirical recommendations of Price et al. (2015) and Zvielli et al. (2015), and the use of personalized stimuli (Christiansen, Mansfield, et al., 2015) to the alcohol VPT in order to examine whether these procedural and analytical changes led to improvements in internal consistency (within both image pairs and tasks) and test–retest reliability. We also examined cross-sectional associations of attentional bias with alcohol consumption and craving. We examined these associations in social drinkers as these individuals also experience craving, and a previous meta-analysis (Field, Munafò, et al., 2009) has demonstrated a link between attentional bias and craving irrespective of drinking status. First, we reanalyzed existing data from three published studies (Field et al., 2007; Field, Duka, et al., 2009; Schoenmakers, Wiers, & Field, 2008) to provide internal consistency estimates (not previously reported) and examine whether different outlier cutoffs influenced these estimates. Then, in Study 1 we examined the internal consistency and test–retest reliability of a standard VPT and VPT recommended by Price et al. (2015) using general alcohol-related cues. We hypothesized that internal consistency estimates would be greater for the recommended task compared with the standard task. In Study 2 we examined the internal consistency and test–retest reliability of the recommended VPT, with general and personalized alcohol-related cues and concurrent eye tracking. We hypothesized that personalized cues would lead to greater internal consistency estimates than general cues, and internal consistency would be further improved by eye-tracking. In each study we also hypothesized that attentional bias measures computed from winsorized RTs and bottom-only probe trials would provide greatest internal consistency.

## Method

### Data Reduction and Analyses

For the outlier removed in all studies (preexisting and new) we conducted three different procedures. For the 2 *SD* procedure we removed all individual RTs that were faster than 200 ms and slower than 2,000 ms and then 2 *SD*s above or below the individual mean. For the 3 *SD* procedure we removed all RTs <200 and >2,000 and then those that were 3 *SD*s above or below the mean. For winsorized outlier removal we rescaled values outside of 1.5 interquartile ranges from the Tukey hinges (25th and 75th percentile) of the full RT distribution of all individuals to the last valid value (Price et al., 2015). We also conducted the median absolute deviation (MAD) method of outlier removal (Leys et al., 2013). The MAD method involves calculation of the median value of the individual’s RT distribution and subtracting this from each RT to create a series of absolute values; the median of these values is then multiplied by 1.4826 to calculate the MAD. The MAD was then multiplied by a value of 3. Upper median and lower cutoffs [median ± (MAD × 3)] are then computed and removed. Note that we did not preregister our decision to include MAD as an outlier removal technique for our new data. Attentional bias scores were created for each picture pair by computing mean RTs on each trial type (congruent and incongruent) then subtracting congruent from incongruent RTs (mean^incongruent^ – mean^congruent^), so that larger positive scores were indicative of increased attentional bias.

We also computed TL-BS by matching temporally contiguous pairs of congruous and incongruous trials within the VPT for each subject [“RT 1st Incongruent Trial – RT 1st Congruent Trial,” “RT 2nd Incongruent Trial – RT 2nd Congruent Trial,” and so on]. We conducted TL-BS on winsorized data without removing any RTs more than five trials apart, to ensure the larger number of trials were available for our reliability estimates. This provided us with a maximum of 64 individual bias scores. From these individual bias scores we calculated mean TL-BS positive (mean of all bias scores >0 ms per participant), mean TL-BS negative (mean of all bias scores <0 ms per participant), peak TL-BS positive (largest individual bias score >0 ms), peak TL-BS negative (largest individual bias score <0 ms), and TL-BS variability (the sum of distances between all individual bias scores/number of scores; Zvielli et al., 2015).^2^

For internal consistency estimates we computed McDonald’s ω because Cronbach’s alpha often underestimates internal consistency (Sijtsma, 2009), and many have argued for its use be abandoned (Peters, 2014). For test–retest reliability we computed the intraclass correlation coefficient (ICC) using a two-way random effects model with absolute agreement. In line with Price et al. (2015) we report the single measurement which is an indicator of the reliability if only one assessment point was used, and also the combined measure which reflects the internal consistency of bias scores across the time points. We also reported Pearson’s correlation between the two time points (a more common measure of test–retest reliability), to allow direct comparisons with previously published studies in this area (e.g., Emery & Simons, 2015; Marks et al., 2014). Across each study we used the total bias score with greatest internal consistency to assess cross sectional associations with individual differences in alcohol consumption and craving. Finally we used the cocran r-package when making comparisons based on our internal consistency estimates (Diedenhofen & Musch, 2016).

### Analyzing Internal Consistencies of Preexisting Data

To examine the internal consistency of the VPT we reanalyzed the data from three studies published by our laboratory. Two studies examined attentional bias to alcohol-related (Field et al., 2007; Schoenmakers et al., 2008) and one to smoking-related cues (Field, Duka, et al., 2009). Schoenmakers et al. (2008) examined attentional bias following ingestion of a placebo beverage and an alcoholic beverage in heavy drinkers, and we provide internal consistency estimates for both conditions (this also allows comparisons with Ataya et al. (2012) who reported estimates from alcohol “priming” studies, which were also low <.34). Field et al. (2007) examined attentional bias before and after attentional bias modification in heavy drinkers: here we provide reliability estimates for the baseline session only as it is reasonable to assume attentional bias modification may influence reliability estimates. Finally, Field, Duka, et al. (2009) examined attentional bias to smoking cues before and after attentional bias modification: again, we examined internal consistencies at baseline only. We decided to include estimates of smoking-related internal consistency as Field and Christiansen (2012) demonstrated internal consistencies to smoking-related images should be greater due to more homogenous images, compared with alcohol-related cues. As discussed, there was considerable variability in the task parameters which allowed us to examine whether internal consistency was greater with a larger number of images (30; Field et al., 2007), using different stimulus presentation durations (500 ms vs. 50 ms; Field, Duka, et al., 2009) and when intoxicated (Schoenmakers et al., 2008).

### Overview of Findings From Preexisting Data

Findings for internal consistency for each study, using different outlier removal techniques are presented in Table 1. To summarize, across the three studies internal consistency estimates did not reach acceptable levels (>.70). Estimates were greater when a larger number of picture pairs were used (30 picture pairs). To further investigate this we also examined internal consistencies from the same data sets when randomly selecting eight and 14 picture pairs, we chose eight and 14 to make direct comparisons with the data in Studies 1 and 2 below (8 picture pairs) and Christiansen, Mansfield, et al. (2015; 14 picture pairs). Estimates were also larger for longer stimulus presentations (500 ms vs. 50 ms). We did not observe evidence that estimates were greater for smoking-related images compared with alcohol-related images.[Table-anchor tbl1]

We note that our estimates of trial-level attentional bias scores (Table 2) are consistent with previous observations from tobacco smokers that attentional bias is not a stable construct and considerable variability in bias scores occurs within the task. Furthermore, mean positive bias scores were generally a greater distance from 0 ms than mean negative bias scores suggesting the presence of attentional bias within the task, but this bias may be obscured if one relies on conventional attentional bias scores.[Table-anchor tbl2]

Finally, there was no evidence that any outlier removal technique led to improved internal consistencies across the studies. We examined whether overall attentional bias was present in each study, using the most reliable outlier removal technique (see Table 1). The presence of a positive bias toward substance cues is inconsistently seen. There were significant biases toward smoking cues irrespective of stimulus presentation duration and following alcohol and placebo intoxication, but only when a smaller number of images were used to calculate reliability and bias estimates.

Therefore, to briefly summarize, our reanalyses of existing data suggests that the internal consistency of the VPT for alcohol- and smoking-related cues is inadequate, despite differing task parameters. These findings support observations by Ataya et al. (2012), who also reported poor internal consistency estimates in VPTs used by their laboratory. Below, we report on two new studies which aimed to include personalized stimuli and different variations of the VPT task based on Price et al. (2015). The design, hypotheses, statistical power justification and analyses were preregistered on Open Science Framework prior to data collection (https://osf.io/gb5fz/).^3^

### Current Data

#### Participants

Participants in each study were recruited from the University of Liverpool and local community. In order to take part, participants had to drink alcohol on a regular basis (at least once per week). Participants were excluded if they had a current or previous diagnosis of a substance use disorder, due to ethical considerations (exposure to substance-related cues could evoke craving, which could be problematic in this population). And because our primary interest was the reliability of these tasks in participants without substance use disorder. The studies were approved by the local ethics committee at the University of Liverpool.

#### Questionnaires

##### Timeline Follow-Back (TLFB)

Participants completed 1-week retrospective recalls of their alcohol consumption in United Kingdom units (1 unit = 8 g pure alcohol), on a day-by-day basis. They were provided with an easy-to-follow guide of typical alcoholic drinks and their units, to ensure accurate estimations. The TLFB (Sobell & Sobell, 1992) is considered to be reliable over short periods and demonstrates considerable stability over time (Carey, Carey, Maisto, & Henson, 2004).

##### Approach and Avoidance of Alcohol Questionnaire (AAAQ)

The AAAQ (McEvoy, Stritzke, French, Lang, & Ketterman, 2004) is a self-report measure of craving, using a 14-item scale. It has three subscales: Inclined/Indulgent, Obsessed/Compelled, and Resolved/Regulated. It has good psychometric properties, however studies have suggested a two-factor structure of approach and avoidance dimensions (Klein et al., 2007).

##### VPT(s)

We based the VPTs on those presented in Price et al. (2015). In the standard version of the task the picture pairs were presented on the left and right of the screen followed by a probe (the letter “E” or “F”) and participants had to respond to the location of the probe. In the recommended version the picture pairs were presented at the top and bottom of the screen followed by the probe, and, in this case, participants had to respond to the content of the probe (e.g., press the E key if they saw E, press the F key if they saw F). These is an important distinction between responding to the content rather than location of the probe, simply responding to the location can be interpreted as perceiving the cue on the left or not perceiving the cue on the right, for example. Therefore, responding to the content of the cue should overcome this issue and presumably lead to more reliable bias estimates. We note that the majority of studies now use VPTs which require responding to content rather than location, so this may no longer be “standard,” however, our aim was to compare a task and outlier removal techniques which are empirically recommended to those which have been used previously.

In both tasks trials began with the presentation of a fixation cross (+) for 500 ms. In the standard version this appeared in the direct center of the screen, whereas in the recommended version this appeared in the space at the top of the screen (where the top image would be presented). Following this, the picture pairs would be presented for 500 ms, these images would then be removed from display, and immediately followed by presentation of the probe until a response was made. Each task had 10 (control-control) practice trials, followed by 160 trials, of which each alcohol-control picture pair was presented 128 times, and control-control picture pairs were presented 32 times. The probe appeared with equal frequency in place of the alcohol and control images in the alcohol-control pairings, and an equal number of times on the left and right/top and bottom depending on the task. Each task took approximately 10 min to complete. Note, there is considerable heterogeneity in previously published studies using the VPT for alcohol; for example, our previous assessment of reliability did not include control-control images (Christiansen, Mansfield, et al., 2015), and had fewer trials (68) but a larger number of picture pairs (14). Other studies have used a larger number of trials (252; (Emery & Simons, 2015), included control-control comparison (Field, Mogg, Zetteler, & Bradley, 2004), and varied stimulus presentation durations (Field et al., 2004). As such, there is no agreed protocol for assessing attentional bias using the VPT.

##### Images

Each task had eight alcohol-related and control picture pairs. General alcohol images were taken from our previous studies (Field, Mogg, Mann, Bennett, & Bradley, 2013; Jones et al., 2012) and depicted images such as of a model holding a bottle of beer or a pen to their lips, or a stack of beer crates or books. For the personalized images we used a selection of the images from Christiansen, Mansfield, et al. (2015). We used different control images for the control-control comparisons to prevent habituation to the images. All images were 140 mm × 90 mm. Distance between images was 75 mm in the recommended task and 95 mm in the standard task.

## Study 1: A Comparison Between the Recommended Task and Standard Task Using General Cues

### Participants

Sixty-seven participants (26 male) were recruited with a mean age of 25.08 years (6.53) years. Fifty-seven participants were retained at Time 2 (24 male, mean age 24.82 6.26). The average number of days between sessions was 7.84 ± 1.75. Participants consumed 23.72 (17.81) weekly units of alcohol at Time 1 and 23.91 (15.36) units of alcohol at Time 2; there was no significant difference in units consumed between the two time points, *t*(56) = 0.216, *p* = .830, *d* = −0.029, 95% CI [−0.288, 0.231]. Mean scores on craving subscales were as follows (Time 1: inclined = 3.78 ± 1.76, obsessed = 0.91 ± 1.17, avoidant = 1.25 ± 1.13; Time 2: inclined = 3.74 ± 1.92, obsessed = 0.95 ± 1.15, avoidant = 1.41 ± 1.27). A 3 (subscale) × 2 (time) repeated measures analysis of variance (ANOVA) demonstrated a significant main effect of subscale, *F*(1.55, 83.61) = 160.54, *p* < .001, but no significant effect of time, *F*(1, 54) = 0.13, *p* = .721 or Time × Subscale interaction, *F*(1.49, 80.50) = 0.16, *p* = .788 suggesting craving scores differed on the subscales, but did not change over time.

### Procedure

Participants attended the laboratory and provided informed consent before completing the TLFB and AAAQ. They then completed the standard VPT and recommended VPT, the order of which was counterbalanced across participants. Following completion of the tasks participants left the laboratory and returned between 7 and 14 days later. Upon their return they completed a second TLFB, AAAQ, standard, and recommended VPT (presentation of VPTs was counterbalanced across time and participants) before being thanked and debriefed. Each session lasted approximately 25 min and participants were given course credits. In the standard task of Study 1 we analyzed RTs for the probe occurring on the left side only, to provide a comparison with bottom-only trials in the recommended version.

### Results

#### Internal consistency and test–retest reliability

Internal consistency and test–retest reliability estimates of alcohol attentional bias scores are shown in Table 3. Across both tasks (standard vs. recommended), procedural variables (below only vs. above and below/left only vs. left and right) and outlier estimation technique (2 *SD* vs. 3 *SD* vs. winsorized) the internal consistency was poor. No estimate approached the threshold for acceptable internal consistency (.70), across time points. However, combined winsorized data from probes appearing behind the bottom image in the recommended task had the greatest internal consistency, but this was not significantly greater than the second greatest (winsorized above and below: *t*(55) = 0.046, *p* = .96).[Table-anchor tbl3]

#### Within-subject variability

Mean and peak TL-BS measures and ICC estimates are displayed in Table 4. Mean measures (both positive and negative) offered improved test–retest reliability than peak estimates and variability, with negative TL-BS mean scores providing the greatest reliability (ICC = .434). Reliability estimates from the recommended task were generally superior to those from the standard version. Furthermore, the estimates for negative mean TL-BS scores were greater than estimates from global bias scores, irrespective of outlier removal strategy.[Table-anchor tbl4]

#### Overall attentional bias and associations with alcohol consumption/craving

Mean attentional bias at Time 1 was 5.20 (28.43) ms and at Time 2 was −4.39 (28.92) ms. Neither was significantly different from 0 ms (Time 1: *t*(66) = 1.50, *p* = .139, *d* = 0.183; Time 2 = *t*(54) = −1.13, *p* = .265, *d* = −0.152). There was weak statistical evidence that attentional bias decreased over time, *t*(54) = 1.98, *p* = .053, *d* = 0.266.

At Time 1 there was no significant correlation between attentional bias and units of alcohol consumed (*r* = −.154, *p* = .214) or craving subscales (*r*s < −.212, *p*s > .084). At Time 2 there was no significant correlation between attentional bias and alcohol consumption (*r* = .064, *p* = .640) or craving subscales (*r*s < .108, *p*s > .442).

#### Associations between trial-level biases and alcohol consumption/craving

Mean negative bias scores on the recommended task had the greatest test–retest reliability. At Time 1 there was no significant association with units consumed (*r* = .168, *p* = .173). There were significant associations with both inclined (*r* = .358, *p* = .003) and obsessed subscales (*r* = .250, *p* = .041), but no significant association with the avoidant subscale (*r* = .220, *p* = .074). At Time 2 there were no significant associations with units consumed (*r* = .001, *p* = .992) or craving subscales (*r*s < −.089, *p*s > .520).

## Study 2: A Comparison Between Personalized and General Cues on the Recommended Task, With Eye Movements

### Participants

We recruited 46 individuals (35 female), with an average age of 21.35 (3.98). We retained 42 participants (32 female) at Time 2. The average number of days between sessions was 8.10 ± 2.15. Participants drank 19.93 (9.97) units of alcohol per week at Time 1 and 16.50 (10.27) units of alcohol at Time 2. There was no significant difference in alcohol consumption between the two time points, *t*(41) = 1.63, *p* = .111, *d* = .251. When asked to indicate their preferred alcoholic beverage six (13.0%) participants chose beer, 12 (26.2%) chose wine, six (13.0%), chose cider, and 22 (47.8%) chose vodka. Mean scores on craving subscales were as follows (Time 1: inclined = 3.61 ± 1.77, obsessed = 0.63 ± 0.91, avoidant = 1.12 ± 1.17; Time 2: inclined = 3.06 ± 1.90, obsessed = 0.55 ± 1.05, avoidant = 1.20 ± 1.45). A 3 (subscale) × 2 (time) repeated measures ANOVA demonstrated a significant main effect of subscale, *F*(1.69, 69.10) = 121.58, *p* < .001, but no significant effect of time, *F*(1, 41) = 3.23, *p* = .080 or Time × Subscale interaction, *F*(1.39, 56.49) = 0.3.29, *p* = .062 suggesting craving scores differed on the subscales, but did not change over time.

### Procedure

Participants attended the laboratory and provided informed consent before completing the TLFB and AAAQ. They then reported their preferred drink out of beer, wine, cider, or vodka (Christiansen, Mansfield, et al., 2015). They then completed a recommended VPT with general alcohol images and a task with alcohol images personalized to their preferred drink (counterbalanced), with concurrent eye tracking. There was no overlap between the general and personalized image sets, to reduce the possibility participants habituated to the images across sessions. Following this they left the laboratory, and returned between 7 and 14 days later. Upon their return they completed a second TLFB, AAAQ, and two VPTs (general stimuli and personalized stimuli, counterbalanced), with concurrent eye tracking. Each session lasted approximately 25 min and individuals were given course credits for their participation. Eye movements were measured using the ASL D6 (Advanced Science Laboratories, Bedford, Massachusetts) eye tracker continuously recording data at 120 Hz.

### Data Reduction and Analysis for Eye Movements

We computed gaze dwell time as the total amount of time (ms) that participants fixated on images, with a fixation defined as a stable eye movement within 1° of visual angle for 100 ms or longer (see previous studies: Jones et al., 2012; Christiansen, Mansfield, et al. (2015)). Bias scores were calculated by subtracting gaze dwell times on neutral images from alcohol images separately for each picture pair.

### Results

#### Internal consistency and test–retest reliability of RT data

Internal consistency and test–retest reliability estimates of alcohol attentional bias scores are shown in Table 5. Across both stimulus sets (personalized vs. general), procedural variables (below only vs. above and below), and outlier estimation technique (2 *SD* vs. 3 *SD* vs. winsorized) the internal consistency was poor. Data from personalized cues with 2 *SD* outliers and above and below probes approached the threshold for acceptable internal consistency (.63), however this was only at Time 1 and was not significantly greater than the second greatest (3 *SD* above and below: *t*(44) = 1.289, *p* = .204).[Table-anchor tbl5]

#### Within-subject variability

For TL-BS estimates, see Table 4. As in Study 1, mean estimates had greater test–retest reliability than peak estimates. The estimates which provided the greatest test–retest reliability were from the negative mean bias score using personalized images (ICC = .446). As in Study 1, this estimate was superior to the estimate obtained from global bias measures, irrespective of outlier removal techniques.

#### Internal consistency and test–retest reliability of eye-movement data

Internal consistency and test–retest reliability estimates of alcohol attentional bias using eye movements are shown in Table 6. As with RT data the internal consistency estimates were poor; the greatest estimates came from personalized cues using all trials (.570), but this still fell short of the cutoff for acceptability. Test–retest reliability was also poor with general alcohol bias demonstrating the greatest reliability.[Table-anchor tbl6]

#### Overall attentional bias (RTs) and associations with alcohol consumption and craving

Mean attentional bias at Time 1 was 3.55 (28.32) ms and at Time 2 was 3.00 (15.65) ms. Neither was significantly different from 0 ms (Time 1: *t*(45) = 0.85, *p* = .400, *d* = 0.125; Time 2 = *t*(41) = 1.24, *p* = .221, *d* = 0.192). Attentional bias did not significantly change over time, *t*(41) = 0.04, *p* = .970, *d* = 0.006. Furthermore, there was no significant difference between attentional bias to personalized cues compared with general cues at either time point (*p*s > .550).

At Time 1 there were no significant correlations between attentional bias and units of alcohol consumed (*r* = −.003, *p* = .984) or craving subscales (*r*s < −.135, *p*s > .372). At Time 2 there were no significant correlations between attentional bias and alcohol consumption (*r* = .004, *p* = .978) or craving subscales (*r*s < −.045, *p*s > .777).

#### Associations between trial-level biases and alcohol consumption/craving

Mean negative bias scores to personalized cues had the greatest test–retest reliability. At Time 1 there was no significant association between mean negative bias and units consumed (*r* = .058, *p* = .701). There were significant associations with both inclined (*r* = .467, *p* = .001) and obsessed subscales (*r* = .345, *p* = .019), but no significant association with the avoidant subscale (*r* = .212, *p* = .157). At Time 2 there were no significant associations with units consumed (*r* = .181, *p* = .251) or craving subscales (*r*s < .199, *p*s > .207).

#### Overall attentional bias (eye movements) and associations with alcohol consumption and craving

Mean attentional bias inferred from gaze dwell times at Time 1 was −1.00 ms (16.00 ms), and at Time 2 was 2.00 ms (19.00 ms). Neither was significantly different from 0 ms (Time 1: *t*(45) = −0.516, *p* = .608, *d* = −0.076; Time 2: *t*(39) = 0.805, *p* = .426, *d* = 0.127). Attentional bias did not significantly change over time, *t*(39) = −0.921, *p* = .363, *d* = −0.146. Personalized cues did not differ significantly from general cues at either time point (*p*s > .375).

Attentional bias was not significantly associated with units consumed at Time 1 (*r* = .201, *p* = .180) or craving subscales (*r*s < .204, *p*s > .174). Similarly, there was no significant association between attentional bias and units consumed (*r* = .152, *p* = .350) at Time 2. However, there was a significant positive association with inclined (*r* = .340, *p* = .032) and obsessed subscales (*r* = .426, *p* = .006) at Time 2. There was no significant association with the avoidant subscale (*r* = .054, *p* = .742).

## Discussion

The aim of this series of studies was to attempt to improve the internal consistency and test–retest reliability of the alcohol/smoking VPT by using recently published empirical recommendations. First, we observed that estimates of internal consistency of VPTs in previously published studies were less than acceptable irrespective of outlier removal techniques. Furthermore, we demonstrated limited support for empirical recommendations in improving psychometric properties of the VPT across all studies, as both internal consistency and test–retest reliabilities were consistently poor.

Our findings contribute to the growing body of evidence which suggests that assessing attentional bias to alcohol (and smoking) using the VPT is unreliable (Ataya et al., 2012). However, these observations are not limited to substance-related cues. Chapman, Devue, and Grimshaw (2017) reviewed internal consistencies across a number of studies examining threatening images, pain-related images, and fearful faces and demonstrated split-half reliabilities ranging from −.22 to .59. Furthermore, they demonstrated reliabilities were only acceptable when cues were presented for short periods (100 ms) suggesting longer time periods such as those regularly used here (500 ms) and in the wider addiction literature (500–2,000 ms) allow attention to be disengaged and reallocated before a probe appears. These findings were corroborated by Waechter, Nelson, Wright, Hyatt, and Oakman (2014), however, they demonstrated direct measures of attention (eye movements) had excellent reliability at longer stimulus durations (5,000 ms).

While personalized stimuli led to greater internal consistency in Study 2, we were unable to replicate previous findings which have demonstrated that using alcohol-related cues based on an individual’s preferred drink improves the internal consistency of the VPT to acceptable levels (Christiansen, Mansfield, et al., 2015). We can speculate as to why we did not replicate these findings. It is possible that the larger number of alcohol-neutral picture pairs (14 vs. 8) in Christiansen, Mansfield, et al. (2015) increased the internal reliability estimate as Cronbach’s alpha has been demonstrated to increase as a function of items in the scale (Tavakol & Dennick, 2011); indeed, we also noted that in Field et al. (2007) reliabilities were close to the acceptable threshold with 30 images (see Table 1) and this declined when a lower number of images was used to estimate internal consistency. Nevertheless, to directly compare across studies we took alpha (.73) from personalized cues from Christiansen, Mansfield, et al. (2015) and used alpha for Study 2 (.61), personalized cues using 2 *SD* outlier removal, and demonstrated no significant difference between the two, *F*(59, 45) = 1.44, *p* = .200.

We also demonstrated poor test–retest reliability across time points in all studies, image type,s and outlier removal, with the greatest reliability demonstrated using trial-level estimates. These findings are similar to those assessing test-retest of cocaine attentional bias (Marks et al., 2014), and anxiety-related words/pictures (Price et al., 2015). While these findings might be attributable to measurement inadequacy, it is also possible that attentional bias demonstrates low stability/state dependence (Hedge, Powell, & Sumner, 2018). Recent theoretical models suggest that attentional bias is sensitive to immediate momentary evaluations, which, in turn, is sensitive to a myriad of internal and environmental factors (Field et al., 2016) which may differ across testing sessions. These observations are supported by Zvielli et al., (2015) who demonstrated phasic bursts of attentional bias within the task, and is supported by our TL-BS which demonstrated improved (but still not acceptable) test–retest reliability in Studies 1 and 2.

We found limited evidence of significant associations between attentional bias and alcohol consumption or craving, unlike previous studies and meta-analyses (Field, Munafò, et al., 2009; Marks et al., 2014). One explanation for this is that these associations *exist* but are obscured by poor psychometric properties of the VPT (Rodebaugh et al., 2016). In support of this, Christiansen and Bloor (2014) demonstrated that personalized cues were predictive of alcohol use but general cues were not using the Stroop task in social drinkers (cf., equivocal findings in dependent drinkers; Fridrici et al., (2013), and in Study 2 we demonstrated tentative evidence of positive associations with craving when using global bias measures. Trial-level bias estimations also demonstrated that as attentional avoidance of alcohol cues (increased negative bias scores) increased in strength, subjective craving reduced in strength. However it is reasonably likely that findings throughout the literature are overstated or there is no meaningful relationship (Christiansen, Schoenmakers, et al., 2015). Christiansen, Mansfield, et al. (2015) did not find any associations with alcohol use/craving when internal consistency was greater than the acceptable threshold (see also Waechter et al., (2014) in social anxiety). Furthermore, a lack of standardized protocol for the VPT allows for researcher degrees of freedom which may artificially inflate associations through “significance chasing” (Ware & Munafò, 2015).

The major implication of these findings is that the poor reliability of the VPT was consistently evident despite numerous attempts at stimuli, analyses, and protocol changes aimed at improving the reliability. Given the widespread use of the VPT in the literature this may have wide-reaching consequences and it is probable that that the VPT is not reliable in nonclinical populations and should not be used as a diagnostic tool (Schmukle, 2005). Furthermore, McNally (2018) suggests that the lack of reliability of the VPT and other attentional measurements is an emerging crisis which may threaten survival of the field. Therefore, a focus on improving reliability is urgently needed to help accurately test theoretical predictions of addiction models (Franken, 2003; Robinson & Berridge, 2001), but also whether attentional bias modification using modified VPTs can lead to robust clinically relevant outcomes (Cox, Fadardi, Intriligator, & Klinger, 2014). Until we can develop robust, reliable RT measures of attentional bias future research should focus on measuring direct attention wherever possible using eye-tracking technology, as this has been demonstrated to show greatest levels of internal consistency and test–retest reliability in other studies (Christiansen, Mansfield, et al., 2015; Waechter et al., 2014). Eye tracking may provide more reliable measures as it is not dependent on manual RTs which are distally related to attentional capture, and can be confounded by intervening emotional processes and response execution (Armstrong & Olatunji, 2012). Furthermore, it also allows for researchers to distinguish different stages of attention (early vs. late) as well as other potentially useful measures, such as latency and direction of initial fixation (Hardman, Scott, Field, & Jones, 2014).

There are limitations to our studies. First, we did not specifically recruit heavy drinkers. It may be that reliability of the VPT will be greater in heavy drinkers and alcohol- dependent patients who are thought to demonstrate more robust attentional bias (Schoenmakers et al., 2010; Townshend & Duka, 2001). However, we note that the average alcohol consumption in our studies suggests the majority of our samples were heavy drinkers. Furthermore, the averages are comparable to the data sets we reanalyzed from Field et al. (2007) and Schoenmakers et al. (2008) who specifically recruited heavy drinkers but did not have acceptable internal consistency. In relation to this, we found limited evidence for the presence of bias using global measures in our newly collected data, and mixed evidence in previous data sets. However, we note that mean positive bias scores were greater than mean negative bias scores using trial-level data across the data sets suggesting biases may exist at periods during the task, but this may be obscured when examining global bias scores. Indeed, similar studies have failed to detect a global bias in nondependent drinkers (Groefsema, Engels, Kuntsche, Smit, & Luijten, 2016; Manchery, Yarmush, Luehring-Jones, & Erblich, 2017). Therefore, future research should further examine the utility of trial-level biases (however, others have suggested limited potential for these indices; Kruijt, Field, & Fox, 2016). Similarly, the absence of overall bias in Studies 1 and 2 may also be attributable to the (lack of) reliability of the VPT to robustly detect these biases rather than an absence in the current samples. Second, we are unable to provide any estimates for alcohol-dependent patients and future research should establish the reliability of the VPT in these samples.

To conclude, in a series of studies we attempted to improve the internal consistency and test–retest reliability of the VPT task for alcohol (and smoking related) using previously published recommendations. Across five data sets (3 preexisting and 2 novel) we did not find adequate internal consistency or test–retest reliability, adding to concerns that the VPT is an unreliable measure of attentional bias for substance-related stimuli.

## Figures and Tables

**Table 1 tbl1:** Reanalysis of Existing Data to Examine Internal Consistency Using Different Outlier Removal Methods

Study	No.	No. pics	2 *SD*	3 *SD*	Win	MAD	BIAS
Field et al. (2007)	60	30	.547	.623	**.652**	.622	−2.15 (27.75)
	60	8	.278	.350	.215	.281	1.00 (37.57)
	60	14	.344	.445	.434	.398	−1.30 (31.81)
Field, Duka, Tyler, and Schoenmakers (2009) 50 ms							
	72	10	.169	.223	.203	−.068	12.99 (27.83)**
Field, Duka, et al. (2009), 500 ms							
	72	10	**.463**	.408	.425	.425	16.51 (40.40)**
Schoenmakers, Wiers, and Field (2008), placebo							
	26	14	**.573**	.562	.242	.303	15.86 (47.54)
	26	8	.424	.453	.212	.345	23.01 (51.18)*
Schoenmakers et al. (2008), alcohol							
	26	14	.573	.562	.583	**.592**	16.89 (49.05)
	26	8	.424	.453	.493	.467	22.16 (53.38)*
*Note.* Values in bold type had greatest internal consistency/test–retest reliability. BIAS = mean bias score in ms across all trials (* *p* < .05. ** *p* < .01 for one-sampled *t* test against 0 ms); MAD = median absolute deviation outlier removal; *SD* = standard deviation outlier removal; Win = winsorized outlier removal.							

**Table 2 tbl2:** Within-Subject Variability in Bias Scores for the Reanalysis of Previous Data (Values Are Means and Standard Deviations)

Existing data	Mean positive	Peak positive	Mean negative	Peak negative	Variability
Field et al. (2007)	107.82 (24.79)	316.70 (74.74)	−53.43 (14.30)	−321.32 (71.59)	148.19 (30.77)
Field, Duka, et al. (2009)					
50 ms	145.70 (38.04)	426.67 (121.20)	−65.53 (23.07)	−408.63 (126.21)	196.29 (47.26)
500 ms	152.43 (41.99)	449.43 (129.59)	−65.36 (26.80)	−387.97 (140.55)	205.71 (55.10)
Schoenmakers et al. (2008)					
Alcohol	125.76 (31.53)	300.08 (90.48)	−51.63 (23.68)	−284.31 (90.80)	164.84 (43.81)
Placebo	100.59 (22.99)	229.00 (56.46)	−51.95 (14.84)	−265.19 (62.49)	142.69 (22.70)

**Table 3 tbl3:** Measures of Internal Consistency and Test–Retest Reliability in Study 1

Data handling	Time 1	Time 2	Combined	ICC^s^	ICC^c^	*r*
Recommended task						
2 *SD*	**.374**	.168	.499	.133	.235	.143
2 *SD* Below/left	.043	.300	.472	.058	.109	.060
3 *SD*	.224	.152	.480	.086	.159	.093
3 *SD* Below/left	.137	.249	.449	−.015	−.031	−.016
Win	.239	.300	.506	.159	.274	.174
Win Below/left	.146	**.343**	**.510**	.047	.090	.049
MAD	.365	.229	.361	.146	.255	.155
MAD Below	.120	.311	.191	.090	.167	.091
Standard task						
2 *SD*	−.083	−.011	.415	.224	.366	.231
2 *SD* Below/left	−.108	.016	.397	.107	.193	.111
3 *SD*	.074	.196	.451	.164	.282	.169
3 *SD* Below/left	.104	−.024	.406	.150	.261	.160
Win	.028	.083	.415	**.285**	**.444**	.302*
Win Below/left	−.007	.136	.397	.002	.004	.002
MAD	−.097	−.070	.031	.176	.300	.179
MAD Below	−.023	−.075	−.036	−.057	−.121	−.057
*Note.* Values in bold type had greatest internal consistency/test–retest reliability. Below/left = trials in which probe appeared behind the bottom or left image only; ICC^s^ = intraclass correlation of single estimate; ICC^c^ = intraclass correlation of the combined estimates; MAD = median absolute deviation outlier removal; *SD* = standard deviation outlier removal; Win = winsorized outlier removal.						
* *p <* .05 (.024).						

**Table 4 tbl4:** Trial-Level Bias Scores and Test–Retest Reliability Estimates for Studies 1 and 2

TL-BS	Time 1 *M*	Time 2 *M*	ICC^s^	ICC^c^	*r*
Study 1 (standard)					
Mean positive	63.33 (17.77)	57.13 (14.04)	.347	.515	.376
Peak positive	195.03 (54.83)	189.00 (46.00)	.142	.249	.143
Mean negative	−33.21 (8.74)	−28.11 (8.51)	.164	.282	.193
Peak negative	−207.54 (51.70)	−184.40 (53.12)	.222	.363	.244
Variability	85.67 (22.54)	80.25 (16.52)	.376	.547	.405
Study 1 (recommended)					
Mean positive	119.64 (28.01)	112.64 (27.60)	.403	.574	.410
Peak positive	363.73 (87.16)	331.46 (83.75)	.190	.320	.205
Mean negative	−58.31 (17.84)	−53.39 (14.56)	**.434**	**.605**	**.451**
Peak negative	−365.41 (96.51)	−353.74 (82.29)	.116	.208	.118
Variability	163.16 (37.01)	153.17 (34.48)	.423	.595	.437
Study 2 (general cues)					
Mean positive	131.22 (33.68)	113.96 (22.98)	.385	.556	.474
Peak positive	390.15 (99.50)	339.12 (66.66)	.123	.220	.155
Mean negative	−68.78 (15.66)	−59.20 (14.16)	.377	.547	.443
Peak negative	−421.91 (89.11)	−342.83 (73.76)	.107	.193	.156
Variability	183.92 (37.56)	156.52 (27.22)	.323	.488	.439
Study 2 (personalized cues)					
Mean positive	133.21 (41.67)	108.50 (22.57)	.286	.445	.432
Peak positive	383.48 (98.99)	310.31 (73.69)	.161	.277	.244
Mean negative	−62.45 (16.26)	−57.63 (14.37)	**.446**	**.617**	**.453**
Peak negative	−374.28 (85.83)	−342.21 (74.58)	.223	.365	.234
Variability	186.68 (45.37)	154.44 (33.21)	.421	.593	.570
*Note.* Time 1 *M* and Time 2 *M* = mean score (standard deviation in brackets); ICCs = intraclass correlation of single estimate; ICCc = intraclass correlation of the combined estimates. Values in bold type had greatest internal consistency/test–retest reliability.					

**Table 5 tbl5:** Measures of Internal Consistency and Test–Retest Reliability From Reaction Times in Study 2

Data handling	Time 1	Time 2	Combined	ICC^s^	ICC^c^	*r*
General images						
2 *SD*	.045	.326	−.023	.072	.135	.072
2 *SD* Below	.200	.139	.314	−.055	−.166	−.055
3 *SD*	.228	**.331**	.118	−.045	−.093	−.045
3 *SD* Below	.267	.267	.018	−.167	−.401	−.175
Win	.245	.292	.105	.003	.006	.003
Win Below	.353	−.080	.115	−.150	−.354	−.158
MAD	−.013	.068	.013	−.040	−.084	−.040
MAD below	.074	−.144	.207	−.075	−.163	−.077
Personalized images						
2 *SD*	**.633**	.142	.440	−.038	−.346	−.045
2 *SD* Below	.205	.225	.260	−.075	−.163	−.077
3 *SD*	.581	.101	.423	.134	.236	.159
3 *SD* Below	.287	.177	.117	.018	.035	.020
Win	.289	.033	.264	**.205**	**.341**	**.213**
Win Below	−.223	.244	.106	.101	.183	.109
MAD	.628	.063	**.457**	.039	.076	.047
MAD Below	.283	.220	.158	−.084	−.184	−.088
*Note.* Values in bold type had greatest internal consistency/test–retest reliability. Below = trials in which probe appeared behind the bottom image only; ICC^s^ = intraclass correlation of single estimate; ICC^c^ = intraclass correlation of the combined estimates; MAD = median absolute deviation outlier removal; *SD* = standard deviation outlier removal; Win = winsorized outlier removal.						

**Table 6 tbl6:** Measures of Internal Consistency and Test–Retest Reliability From Eye Movements in Study 2

Trial type	Time 1	Time 2	Combined	ICC^s^	ICC^c^	*r*
General images						
All trials	−.449	.283	−.039	**.042**	**.081**	**.044**
Below	−.999	−.894	−.999	.027	.053	.027
Personalized images						
All trials	**.360**	**.570**	**.480**	−.052	−.109	−.053
Below	−.711	−.765	−.340	.030	.059	.030
*Note.* Values in bold type had greatest internal consistency/test–retest reliability. Below = trials in which probe appeared behind the bottom image only; ICC^s^ = intraclass correlation of single estimate; ICC^c^ = intraclass correlation of the combined estimates.						

## Footnotes

1 A Google Scholar search for ‘Visual Probe Task’ identifies 406,000 results (as of 08/05/2018).

2 Note, we did not preregister TL-BS analyses. These were recommended by a helpful reviewer during peer review. We were unable to calculate internal consistency estimates for TL-BS scores due to the large number of trials that are removed when using <0 ms and >0 ms as required.

3 There was a major deviation from our preregistration in which we use McDonald’s ω as our measure of internal consistency (discussed in the Method section).

## References

[c44] Armstrong T., & Olatunji B. O. (2012). Eye tracking of attention in the affective disorders: a meta-analytic review and synthesis. Clinical Psychology Review, 32, 704–723. 10.1016/j.cpr.2012.09.004 23059623 PMC3556338

[c1] Ataya A. F., Adams S., Mullings E., Cooper R. M., Attwood A. S., & Munafò M. R. (2012). Internal reliability of measures of substance-related cognitive bias. Drug and Alcohol Dependence, 121, 148–151. 10.1016/j.drugalcdep.2011.08.023 21955365

[c2] Carey K. B., Carey M. P., Maisto S. A., & Henson J. M. (2004). Temporal stability of the Timeline Followback interview for alcohol and drug use with psychiatric outpatients. Journal of Studies on Alcohol, 65, 774–781. 10.15288/jsa.2004.65.774 15700516 PMC2424021

[c3] Chapman A., Devue C., & Grimshaw G. M. (2017). Fleeting reliability in the dot-probe task. Psychological Research. Advance online publication 10.1007/s00426-017-0947-6 29159699

[c4] Christiansen P., & Bloor J. F. (2014). Individualised but not general alcohol Stroop predicts alcohol use. Drug and Alcohol Dependence, 134, 410–413. 10.1016/j.drugalcdep.2013.10.021 24275612

[c5] Christiansen P., Mansfield R., Duckworth J., Field M., & Jones A. (2015). Internal reliability of the alcohol-related visual probe task is increased by utilising personalised stimuli and eye-tracking. Drug and Alcohol Dependence, 155, 170–174. 10.1016/j.drugalcdep.2015.07.672 26239377

[c6] Christiansen P., Schoenmakers T. M., & Field M. (2015). Less than meets the eye: Reappraising the clinical relevance of attentional bias in addiction. Addictive Behaviors, 44, 43–50. 10.1016/j.addbeh.2014.10.005 25453782

[c45] Cox W. M., Fadardi J. S., Intriligator J. M., & Klinger E. (2014). Attentional bias modification for addictive behaviors: clinical implications. CNS Spectrums, 19, 215–224. 10.1017/S1092852914000091 24642267

[c7] Diedenhofen B., & Musch J. (2016). cocron: A web interface and R package for the statistical comparison of Cronbach’s alpha coefficients. International Journal of Internet Science, 11, 51–60.

[c8] Emery N. N., & Simons J. S. (2015). Mood & alcohol-related attentional biases: New considerations for gender differences and reliability of the visual-probe task. Addictive Behaviors, 50, 1–5. 10.1016/j.addbeh.2015.06.007 26091585 PMC4515404

[c9] Erceg-Hurn D. M., & Mirosevich V. M. (2008). Modern robust statistical methods: An easy way to maximize the accuracy and power of your research. American Psychologist, 63, 591–601. 10.1037/0003-066X.63.7.591 18855490

[c10] Field M., & Christiansen P. (2012). Commentary on Ataya et al. (2012), ‘Internal reliability of measures of substance-related cognitive bias’. Drug and Alcohol Dependence, 124, 189–190. 10.1016/j.drugalcdep.2012.02.009 22386685

[c11] Field M., Duka T., Eastwood B., Child R., Santarcangelo M., & Gayton M. (2007). Experimental manipulation of attentional biases in heavy drinkers: Do the effects generalise? Psychopharmacology, 192, 593–608. 10.1007/s00213-007-0760-9 17361393

[c12] Field M., Duka T., Tyler E., & Schoenmakers T. (2009). Attentional bias modification in tobacco smokers. Nicotine & Tobacco Research, 11, 812–822. 10.1093/ntr/ntp067 19474181

[c50] Field M., & Eastwood B. (2005). Experimental manipulation of attentional bias increases the motivation to drink alcohol. Psychopharmacology, 183, 350–357. 10.1007/s00213-005-0202-5 16235080

[c70] Field M., Mogg K., Mann B., Bennett G. A., & Bradley B. P. (2013). Attentional biases in abstinent alcoholics and their association with craving. Psychology of Addictive Behaviors, 27, 71–80. 10.1037/a0029626 22905898

[c13] Field M., Mogg K., Zetteler J., & Bradley B. P. (2004). Attentional biases for alcohol cues in heavy and light social drinkers: The roles of initial orienting and maintained attention. Psychopharmacology, 176, 88–93. 10.1007/s00213-004-1855-1 15071718

[c14] Field M., Munafò M. R., & Franken I. H. A. (2009). A meta-analytic investigation of the relationship between attentional bias and subjective craving in substance abuse. Psychological Bulletin, 135, 589–607. 10.1037/a0015843 19586163 PMC2999821

[c46] Field M., & Powell H. (2007). Stress increases attentional bias for alcohol cues in social drinkers who drink to cope. Alcohol and Alcoholism, 42, 560–566. 17766316 10.1093/alcalc/agm064

[c15] Field M., Werthmann J., Franken I., Hofmann W., Hogarth L., & Roefs A. (2016). The role of attentional bias in obesity and addiction. Health Psychology, 35, 767–780. 10.1037/hea0000405 27505196

[c16] Franken I. H. A. (2003). Drug craving and addiction: Integrating psychological and neuropsychopharmacological approaches. Progress in Neuro-Psychopharmacology & Biological Psychiatry, 27, 563–579. 10.1016/S0278-5846(03)00081-2 12787841

[c17] Fridrici C., Leichsenring-Driessen C., Driessen M., Wingenfeld K., Kremer G., & Beblo T. (2013). The individualized alcohol Stroop task: No attentional bias toward personalized stimuli in alcohol-dependents. Psychology of Addictive Behaviors, 27, 62–70. 10.1037/a0029139 22747499

[c18] Groefsema M., Engels R., Kuntsche E., Smit K., & Luijten M. (2016). Cognitive biases for social alcohol-related pictures and alcohol use in speficic social settings: An event-level study. Alcoholism: Clinical and Experimental Research, 40, 2001–2010. 10.1111/acer.13165 27511292

[c71] Hardman C. A., Scott J., Field M., & Jones A. (2014). To eat or not to eat. The effects of expectancy on reactivity to food cues. Appetite, 76, 153–160. 10.1016/j.appet.2014.02.005 24530655

[c47] Hedge C., Powell G., & Sumner P. (2018). The reliability paradox: Why robust cognitive tasks do not produce reliable individual differences. Behavior Research Methods, 50, 1166–1186. 10.3758/s13428-017-0935-1 28726177 PMC5990556

[c49] Iacoviello B. M., Wu G., Abend R., Murrough J. W., Feder A., Fruchter E., . . . Charney D. S. (2014). Attention bias variability and symptoms of posttraumatic stress disorder. Journal of Trauma Stress, 27, 232–239. 10.1002/jts.21899 PMC4617532 24604631

[c48] Jones A., Hogarth L., Christiansen P., Rose A. K., Martinovic J., & Field M. (2012). Reward expectancy promotes generalized increases in attentional bias for rewarding stimuli. Quarterly Journal of Experimental Psychology, 65, 2333–2342. 10.1080/17470218.2012.686513 22631033

[c21] Klein A. A., Stasiewicz P. R., Koutsky J. R., Bradizza C. M., & Coffey S. F. (2007). A psychometric evaluation of the Approach and Avoidance of Alcohol Questionnaire (AAAQ) in alcohol dependent outpatients. Journal of Psychopathology and Behavioral Assessment, 29, 231–240. 10.1007/s10862-007-9044-2

[c22] Kruijt A.-W., Field A. P., & Fox E. (2016). Capturing dynamics of biased attention: Are new attention variability measures the way forward? PLoS ONE, 11, e0166600 10.1371/journal.pone.0166600 27875536 PMC5119769

[c51] Leys C., Ley C., Klein O., Bernard P., & Licata L. (2013). Detecting outliers: Do not use standard deviation around the mean, use absolute deviation around the median. Journal of Experimental Social Psychology, 49, 764–766.

[c23] Luehring-Jones P., Louis C., Dennis-Tiwary T. A., & Erblich J. (2017). A single session of attentional bias modification reduces alcohol craving and implicit measures of alcohol bias in young adult drinkers. Alcoholism: Clinical and Experimental Research, 41, 2207–2216. 10.1111/acer.13520 PMC5711540 28992377

[c24] MacLeod C., Mathews A., & Tata P. (1986). Attentional bias in emotional disorders. Journal of Abnormal Psychology, 95, 15–20. 10.1037/0021-843X.95.1.15 3700842

[c25] Manchery L., Yarmush D. E., Luehring-Jones P., & Erblich J. (2017). Attentional bias to alcohol stimuli predicts elevated cue-induced craving in young adult social drinkers. Addictive Behaviors, 70, 14–17. 10.1016/j.addbeh.2017.01.035 28161617

[c26] Marks K. R., Pike E., Stoops W. W., & Rush C. R. (2014). Test-retest reliability of eye tracking during the visual probe task in cocaine-using adults. Drug and Alcohol Dependence, 145, 235–237. 10.1016/j.drugalcdep.2014.09.784 25456573 PMC4268011

[c27] McEvoy P. M., Stritzke W. G. K., French D. J., Lang A. R., & Ketterman R. (2004). Comparison of three models of alcohol craving in young adults: A cross-validation. Addiction, 99, 482–497. 10.1111/j.1360-0443.2004.00714.x 15049748

[c28] McNally R. J. (2018). Attentional bias for threat: Crisis or opportunity? Clinical Psychology Review. Advance online publication 10.1016/j.cpr.2018.05.005 29853421

[c52] Miller M. A., & Fillmore M. T. (2010). The effect of image complexity on attentional bias toward alcohol-related images in adult drinkers. Addiction (Abingdon, England), 105, 883–890. 10.1111/j.1360-0443.2009.02860.x PMC2922935 20148790

[c29] Parsons S. (2018a). Moving forward with questions of process and procedure in cognitive bias modification research: Three points of consideration [preprint]. PsyArXiv. 10.31234/osf.io/k3vxc

[c30] Parsons S. (2018b, 6 4). Visualising two approaches to explore reliability-power relationships [preprint]. PsyArXiv. 10.17605/OSF.IO/QH5MF

[c31] Peters G. Y. (2014). The alpha and the omega of scale reliability and validity: Why and how to abandon Cronbach’s alpha. The European Health Psychologist, 16, 56–69.

[c60] Price R. B., Kuckertz J. M., Siegle G. J., Ladouceur C. D., Silk J. S., Ryan N. D., . . . Amir N. (2015). Empirical recommendations for improving the stability of the dot-probe task in clinical research. Psychol Assess, 27, 365–376. 10.1037/pas0000036 25419646 PMC4442069

[c32] Robinson T. E., & Berridge K. C. (2001). Incentive-sensitization and addiction. Addiction, 96, 103–114. 10.1046/j.1360-0443.2001.9611038.x 11177523

[c33] Rodebaugh T. L., Scullin R. B., Langer J. K., Dixon D. J., Huppert J. D., Bernstein A., . . . Lenze E. J. (2016). Unreliability as a threat to understanding psychopathology: The cautionary tale of attentional bias. Journal of Abnormal Psychology, 125, 840–851. 10.1037/abn0000184 27322741 PMC4980228

[c57] Schmukle S. C. (2005). Unreliability of the dot probe task. European Journal of Personality, 19, 595–605. 10.1002/per.554

[c58] Schoenmakers T., de Bruin M., Lux I. F., Goertz A. G., Van Kerkhof D. H., & Wiers R. W. (2010). Clinical effectiveness of attentional bias modification training in abstinent alcoholic patients. Drug and Alcohol Dependence, 109, 30–36. 20064698 10.1016/j.drugalcdep.2009.11.022

[c35] Schoenmakers T., Wiers R. W., & Field M. (2008). Effects of a low dose of alcohol on cognitive biases and craving in heavy drinkers. Psychopharmacology, 197, 169–178. 10.1007/s00213-007-1023-5 18066535 PMC2493055

[c36] Sijtsma K. (2009). On the use, the misuse, and the very limited usefulness of Cronbach’s alpha. Psychometrika, 74, 107–120. 10.1007/s11336-008-9101-0 20037639 PMC2792363

[c37] Sobell L. C., & Sobell M. B. (1992). Timeline follow-back, A technique for assising self-reported alcohol consumption In Litten R. Z. & Allen J. P. (Eds.), Measuring alcohol consumption: Psychosocial and biochemical methods (pp. 41–72). Totowa, NJ: Humana Press.

[c54] Tavakol M., & Dennick R. (2011). Making sense of Cronbach's alpha. International Journal of Medical Education, 2, 53–55. 10.5116/ijme.4dfb.8dfd 28029643 PMC4205511

[c55] Townshend J., & Duka T. (2001). Attentional bias associated with alcohol cues: differences between heavy and occasional social drinkers. Psychopharmacology, 157, 67–74. 10.1007/s002130100764 11512045

[c38] Waechter S., Nelson A. L., Wright C., Hyatt A., & Oakman J. (2014). Measuring attentional bias to threat: Reliability of dot probe and eye movement indices. Cognitive Therapy and Research, 38, 313–333. 10.1007/s10608-013-9588-2

[c39] Ware J. J., & Munafò M. R. (2015). Significance chasing in research practice: Causes, consequences and possible solutions. Addiction, 110, 4–8. 10.1111/add.12673 PMC4962906 25040652

[c40] Werthmann J., Jansen A., & Roefs A. (2015). Worry or craving? A selective review of evidence for food-related attention biases in obese individuals, eating-disorder patients, restrained eaters and healthy samples. The Proceedings of the Nutrition Society, 74, 99–114. 10.1017/S0029665114001451 25311212

[c41] Zimmerman D., & Zumbo B. (2015). Resolving the issue of how reliability is related to statistical power: Adhering to mathematical definitions. Journal of Modern Applied Statistical Methods, 14, 9–26.

[c42] Zvielli A., Amir I., Goldstein P., & Bernstein A. (2016). Targeting biased emotional attention to threat as a dynamic process in time. Clinical Psychological Science, 4, 287–298. 10.1177/2167702615588048

[c43] Zvielli A., Bernstein A., & Koster E. H. W. (2015). Temporal dynamics of attentional bias. Clinical Psychological Science, 3, 772–788. 10.1177/2167702614551572

